# Effects of meal preparation training on body weight, glycemia, and blood pressure: results of a phase 2 trial in type 2 diabetes

**DOI:** 10.1186/1479-5868-9-125

**Published:** 2012-10-17

**Authors:** Kaberi Dasgupta, Samantha Hajna, Lawrence Joseph, Deborah Da Costa, Stavroula Christopoulos, Rejeanne Gougeon

**Affiliations:** 1 Department of Medicine, McGill University, Montreal, QC, Canada; 2 Department of Epidemiology, Biostatistics and Occupational Health, McGill University, Montreal, QC, Canada; 3 Division of Clinical Epidemiology, Department of Medicine, McGill University Health Centre, 687 Pine Avenue West, V-Building (V1.08), Montreal, Canada

**Keywords:** Body weight, Physical activity, Patient education, Risk factors, Type 2 diabetes

## Abstract

**Background:**

Modest reductions in weight and small increases in step- related activity (e.g., walking) can improve glycemic and blood pressure control in type 2 diabetes mellitus (DM2). We examined changes in these parameters following training in time- efficient preparation of balanced, low- energy meals combined with pedometer- based step count monitoring.

**Methods:**

Seventy- two adults with DM2 were enrolled in a 24- week program (i.e., 15 three- hour group sessions). They prepared meals under a chef’s supervision, and discussed eating behaviours/nutrition with a registered dietitian. They maintained a record of pedometer- assessed step counts. We evaluated changes from baseline to 24 weeks in terms of weight, step counts, hemoglobin A1c (HbA1c, glycemic control), blood pressure, and eating control ability (Weight Efficacy Lifestyle WEL Questionnaire). 53 participants (73.6%) completed assessments.

**Results:**

There were improvements in eating control (11.2 point WEL score change, 95% CI 4.7 to 17.8), step counts (mean change 869 steps/day, 95% CI 198 to 1,540), weight (mean change −2.2%; 95% CI −3.6 to −0.8), and HbA1c (mean change −0.3% HbA1c, 95% CI −0.6 to −0.1), as well as suggestion of systolic blood pressure reduction (mean change −3.5 mm Hg, 95% CI −7.8 to 0.9). Findings were not attributable to medication changes. In linear regression models (adjusted for age, sex, ethnicity, insulin use, season), a −2.5% weight change was associated with a −0.3% HbA1c change (95% CI −0.4 to −0.2) and a −3.5% systolic blood pressure change (95% CI −5.5 to −1.4).

**Conclusions:**

In this ‘proof of concept’ study, persistence with the program led to improvements in eating and physical activity habits, glycemia reductions, and suggestion of blood pressure lowering effects. The strategy thus merits further study and development to expand the range of options for vascular risk reduction in DM2.

## Introduction

Type 2 diabetes mellitus (DM2) confers a two- to four- fold risk increase for myocardial infarction (MI) and stroke and is a leading cause of renal injury and visual impairment [1]. A net weight loss of 5% or more leads to improvements in both blood pressure and glycemia, [2-6] and there is even evidence for improvements with net weight loss of as little as 2 to 5% in persons with DM2 [2]. Similarly, small increases in walking and other forms of ‘stepping activity’ have the potential to reduce vascular risk through blood pressure lowering, as we [7,8] and others [9] have previously demonstrated.

Weight loss is challenging, however, in a socio- environmental context characterized by shifts in food consumption patterns, from home- prepared regular meals to more erratic, ‘on the run’ intake of ‘supersized’ prepared meals [10-12]. Moreover, increased use of computers in the workplace, communication, and leisure has led to a marked decline in ‘daily steps [13]. Further, there is evidence that individuals with DM2 have greater difficulty losing weight and increasing physical activity levels compared to those without DM2 [14].

Nonetheless, in the ongoing Look AHEAD trial, important weight losses in individuals with DM2 have been achieved through a dietary intervention that incorporates meal replacements and strong behavioural therapy elements [15]. Not all individuals with DM2, however, may be willing to use meal replacements, because of limited variety and flexibility. There is thus a need for development of a variety of strategies to allow for ‘fit’ with preferences.

Through a Phase 2 trial, we aimed to empower adults with DM2 to meet the challenges of improving weight control and increasing step counts in our modern ‘obesogenic’ environment. We developed a program that combined self-monitoring strategies (e.g., weight, step counts) with culinary skills training to apply nutrition- related education. No meal replacement products were employed. We tested the intervention through a pre- intervention/post- intervention design. Specifically, among overweight adults followed for DM2, we aimed to assess the mean weight change during our 24- week intervention, as well as mean changes in step count, glycemic control (hemoglobin A1c, HbA1c), blood pressure, and control of eating. We also sought to quantify relationships between changes in weight and step counts with changes in HbA1c and blood pressure. The findings reported herein provide important ‘proof of concept’ information and justification for further study and development.

## Methods

### Participants

Recruitment (1 April 2009 to 8 May 2010) occurred at hospital-based outpatient clinics affiliated with McGill University (Montreal, Canada; McGill University Health Centre MUHC, Sir Mortimer Davis Jewish General Hospital, St. Mary’s Hospital), through a combination of posters, newsletters, and staff referrals. Requirements for inclusion were age ≥ 18 years; a physician diagnosis of DM2 with regular follow- up; body mass index in the overweight to stage 2 obesity range (i.e., BMI of 25 to 40 kg/m^2^); and ability to communicate and read in English or French. Criteria for exclusion were multiple daily insulin doses (basal insulin permitted); food allergies, intolerances or dietary restrictions; smoking (i.e., past 12 months), and co-morbid illness (e.g., malignancy) or medication (e.g. steroids, sibutramine, orlistat) that could importantly impact body weight during the 24- week intervention period.

### Procedures and investigations

#### Intervention procedure

The main focus of the intervention was on nutritional issues. A series of fifteen core group-based sessions (three hours per session) were delivered over a 24- week period. Each group included 10 to 12 participants. Sessions were held at one of two local grocery stores, in their kitchen workshops. These workshops, normally used for birthday parties and community group meetings, are equipped with a stove, oven, microwave, sink, and several large tables.

At the first session, each participant received a recipe book [16,17] endorsed by the Canadian Diabetes Association (CDA) and detailing its dietary guidelines. They also received a Yamax SW-701 pedometer, [18,19] were instructed in its positioning, and tested it on- site, during a 10- minute indoor group walk. Participants were encouraged to track daily step counts, and progressively increase these by 500 steps/day each week, with an aim of ultimately achieving at least 8,000 to 10,000 steps/day [20-22]. At the beginning of each of the subsequent sessions, the registered dietitian collected a record of step counts since the last session, and weighed each participant.

At all sessions, participants prepared a balanced meal under a professional chef’s supervision, in smaller groups of 3 to 4, using recipes from the book provided. The chef provided guidance on techniques for selecting fresh produce and performing preparatory work (e.g., cutting vegetables on weekends to expedite preparation during the week), as well as actual food preparation techniques (e.g., slicing, steaming, sautéing, etc.).

Meals were ‘self-served’, with guidance from the dietitian with respect to proportions (i.e., half plate vegetables, quarter plate grains, quarter plate protein/substitute) and portion sizes (i.e., unlimited vegetables, grains filling one’s ‘cupped hand’, protein portion the size of palm) [1]. An interactive nutritional education discussion with the dietitian followed [16,17]. Participants were encouraged to prepare a similar meal at home, and to try other recipes from the book provided.

#### Investigations

At baseline and following the intervention, participants underwent evaluations at the McGill University Health Centre. Demographic information and medication (i.e., antihypertensive and antihyperglycemic medications) use were evaluated. Direct measures included height (without shoes SECA 214 stadiometer), body weight (light clothes, without shoes, post-void, calibrated SECA 882 electronic scale), and circumference of waist (standing position, midway between the lateral lower ribs and the iliac crests after a moderate expiration) and hip (widest level, over the greater trochanters) [23]. Systolic blood pressure (SBP) and diastolic blood pressure (DBP) (two consecutive measurements were taken and averaged for each, Omron HEM-747 IC), and HbA1c (Bio-Rad Variant II) were evaluated. HbA1c, reported as the proportion of hemoglobin molecules that are glycosylated, provides an indication of ambient glucose levels over a two to three month period, the average life span of a red blood cell.

Dietary intake information was derived from a self-administered Food Frequency Questionnaire [24] that assessed total energy, salt, carbohydrate, protein, total fat and saturated fat intakes. Hours dedicated to meal preparation on weekdays and weekend days were queried. Self- perceived cooking ability was evaluated through a series of questions used in a previous study [25]. Control of eating behaviour was evaluated using the Weight Efficacy Lifestyle (WEL) Questionnaire [26,27]. The 20-item WEL instrument scores each item on a 10-point Likert scale (0 to 9), where a higher score is indicative of a greater ability to avoid eating under various circumstances. The five circumstances under which ability to resist eating is assessed include 1) food availability (e.g., attending a party), 2) negative emotions (e.g., anxiety), 3) physical discomfort (e.g., pain), 4) positive activities (e.g., reading or while watching television), and 5) social pressure (e.g., being unable to refuse an invitation to eat). Readiness for weight change was evaluated using the Weight Stages of Change-Short Form [28,29]. Depressive symptomology was assessed using the Center for Epidemiological Studies Depression Scale.

Daily step counts at baseline were estimated from mean values based on records collected at sessions 1 to 3; final step counts were derived from step counts records collected at sessions 13 to 15.

### Ethics

The study was conducted with approval from Institutional Review Board (IRB) of McGill University and at participating institutions (McGill University Health Centre MUHC, Sir Mortimer Davis Jewish General Hospital, St. Mary’s Hospital). All participants provided written informed consent.

### Statistical methods

Baseline characteristics (means and standard deviations (SD) or proportions, as appropriate) were examined separately for those who did and did not complete final assessments. An implausible energy intake was defined as outside the range of 500 to 3500 kcal/d for women and 800 to 4000 kcal/d for men, and these were excluded [30]. Mean changes with 95% confidence intervals (CI) were computed for weight (i.e., percent change from baseline), BMI, step counts, SBP, DBP, HbA1c, dietary intakes (total energy, macronutrient proportion, fibre, sodium), WEL scores and subscale scores, time dedicated to meal preparation, and eating- out frequency. The proportion achieving a 5% or greater weight reduction was computed overall and for those who attended 75% or more of sessions.

Linear regression analysis was used to assess for associations between changes in weight from baseline with corresponding changes in SBP, DBP, and HbA1c. A similar approach was used to assess for associations between changes in step counts and changes in SBP, DBP, and HbA1c. Potential confounders/co-variates considered for inclusion in these models included demographic factors and baseline characteristics. Final models were adjusted for age, sex, ethnocultural background, insulin use (indicator of diabetes ‘severity’), and season (spring/summer vs. fall/winter) [21]. Sensitivity analyses were conducted wherein those with medication changes were excluded (i.e., antihypertensive medications for blood pressure models, antihyperglycemic medications for HbA1c models).

Although changes in weight and changes in step count may not be independent (e.g., increases in step counts may enhance weight loss), we assessed the effects of adjusting for changes in step counts in models examining associations of changes in weight with changes in SBP, DBP, and HbA1c. Similarly, we report the effects of adjusting for weight change in models examining relationships between changes in step counts with changes in SBP, DBP, and HbA1c. These analyses were conducted to discern the independent effects of changes in weight and changes in step counts. Data were analyzed using SAS 9.2 (SAS Institute Inc., Cary, NC, USA).

To account for potential effects of those who did not present for final assessments, the mean weight change was re- computed in two sensitivity analyses: (i) carrying baseline weights forward and (ii) carrying forward the weight recorded at last session attended. Multiple imputations were examined but are not reported herein because they did not substantially alter the final estimates.

## Results

### Participants

Among 112 candidates who underwent preliminary assessment of eligibility, 74 were interested and potentially eligible, 72 completed baseline assessment procedures, and 53 completed final evaluations. Among the 19 who did not complete the final assessment, more than half reported time- related barriers (i.e., 6 work- related; 7 unspecified time constraints). Other reasons included unspecified health issues (2), lack of interest in content (3), and moving out of the city (1). Those who did not complete final assessments were (Table 1) younger, had a higher BMI, were more frequently men, were less often on insulin, reported greater ability to withstand eating pressures (Table 2), and dedicated roughly one hour per day to meal preparation.

**Table 1:** Socio- demographic characteristics, clinical measures, and medications at baseline

	**Final Assessement Attended n=53** [^a^]	**Final Assessment Not Attended (n=19)** [^b^]
Socio-demographic Characteristics		
Age, years, Mean (SD)	59.7 (10.6)	57.2 (9.9)
Women, No. (%)	36 (67.9)	9 (47.4)
Europid, No. (%)	40 (75.5)	15 (79.0)
Born in Canada, No. (%)	27 (50.9)	10 (52.6)
Ever smoked, No. (%)	27 (50.9)	8 (42.1)
Income < $50,000, No. (%)	29 (56.9)	6 (33.3)
Employed, [^c^] No. (%)	27 (50.9)	15 (79.0)
Education (>high school), No. (%)	42 (79.3)	16 (84.2)
Living Alone, No. (%)	19 (35.9)	3 (15.8)
Clinical Measures		
Weight, kg, Mean (SD)	88.3 (14.6)	100.3 (16.3)
BMI, kg/m^2b^, Mean (SD)	33.1 (5.0)	35.4 (5.3)
Systolic blood pressure, mm Hg, Mean (SD)	133.4 (12.5)	137.8 (13.2)
Diastolic blood pressure, mm Hg, Mean (SD)	80.4 (9.0)	83.6 (8.6)
HbA1c, %, Mean (SD)	8.0 (1.7)	8.2 (1.7)
Years with diabetes, Mean (SD)	8.5 (8.7)	8.6 (6.2)
Medication Use		
*Antihypertensive agents, No. (%)*		
One	14 (26.9)	5 (26.3)
Two	9 (17.3)	1 (5.3)
More than two	11 (21.2)	2 (10.5)
*Antihyperglycemic agents, No. (%)*		
One oral Agent	22 (42.3)	4 (21.1)
Two oral Agents	14 (26.9)	9 (47.4)
More than two oral agents	4 (7.7)	3 (15.8)
Insulin	13 (25.0)	2 (10.5)

^a^ Income was based on a sample size of 51; Years with diabetes was based on a sample size of 51; Medication use data were based on a sample size of 52.

^b^ Income was based on a sample size of 18; Years with diabetes was based on a sample size of 17 represent the average steps per day taken during weeks 1 to 3; Frequency of eating out (days/week) was based in a sample size of 8 and represents the average frequency of eating out during weeks 1 to 3.

^c^ Compared to individuals who are unemployed or on sick leave.

**Table 2:** Step counts, dietary intakes, and psychobehavioural characteristics at baseline

	**Final Assessment Attended (n=53)** [^d^]	**Final Assessment Not Attended (n=19)** [^e^]
Step Counts, steps/day, Mean (SD)	7,272 (2,777)	6,408 (2,923)
Dietary Intake		
Energy, kcal/day, Mean (SD)	2,024 (839)	1,576 (486)
Protein, % of total energy intake, [^f^] Mean (SD)	19.2 (3.7)	20.7 (5.5)
Carbohydrate, % of total energy intake^c^, Mean (SD)	44.3 (6.8)	40.6 (8.2)
Fat, % of total energy intake^c^, Mean (SD)	36.7 (6.1)	37.9 (6.1)
Saturated fat, % of total energy intake^f^, Mean (SD)	11.1 (2.3)	11.3 (2.2)
Fibre, g/day, Mean (SD)	21.2 (10.3)	15.0 (4.4)
Sodium, mg/day, Mean (SD)	2,903 (1255)	2,277 (714)
Alcohol Consumption (< once per week), No. (%)	43 (81.1)	15 (79.0)
*Weight Efficacy Life-Style Scores, [*^*g*^*] Mean (SD)*		
Availability Subscale	20.3 (7.8)	22.2 (5.1)
Negative Emotions Subscale	20.2 (9.1)	20.9 (8.4)
Physical Discomfort Subscale	25.4 (7.3)	27.1 (5.3)
Positive Activities Subscale	25.1 (7.6)	26.8 (4.6)
Social Pressure Subscale	23.9 (7.5)	25.8 (4.8)
Total WEL Score	115.0 (32.9)	122.8 (22.6)
*Hours/day spent cooking and washing up, Mean (SD)*		
Weekdays	1.5 (1.0)	0.9 (0.8)
Weekend days	1.7 (1.2)	1.1 (1.0)
Self-perception as competent cook [^h^] ^†^, No. (%)	21 (39.6)	10 (52.6)
Frequency of eating out (days/week), Mean (SD)	1.9 (1.8)	3.2 (2.7)
*Stage of Change for Weight Change Efforts, No. (%)*		
Pre-contemplation	2 (3.8)	0 (0)
Contemplation	13 (24.5)	4 (21.1)
Action	29 (54.7)	13 (68.4)
Maintenance	9 (17.0)	2 (10.5)
Depressive Symptomology (CES-S score >16), No. (%)	15 (28.3)	6 (31.6)

^d^ Steps counts per day were based on a sample size of 52 and represent the average steps per day taken during weeks 1 to 3; Frequency of eating out (days/week) was based on a sample size of 48 and represents the average frequency of eating out during weeks 1 to 3.

^e^ Steps counts per day were based on a sample size of 14 and represent the average steps per day taken during weeks 1 to 3; Frequency of eating out (days/week) was based in a sample size of 8 and represents the average frequency of eating out during weeks 1 to 3.

^f^ Assuming that 1 gram of protein equals 4 kcal, 1 gram of carbohydrates equals 4 kcal, 1 gram of total fat equals 9 kcal and 1 gram of saturated fat equals 9 kcal.

^g^ A higher score is indicative of a greater ability to resist eating under the given condition. WEL subscale scores range from 0 to 36. The Total WEL Score ranges from 0 to 180.

^h^ Compared to individuals who perceived that they can cook with a recipe, can prepare simple meals, it is not their role to cook or do not know how to cook.

Among the 53 completing final assessments (Tables 1 and 2), three- quarters were Europid, roughly half Canada- born, and nearly 60% were employed. They were on average at the stage 1 level of obesity with abdominal obesity (mean waist circumference (SD) 103 (10.4) cm in women and 109 (11) cm in men; waist to hip 0.88 (0.07) in women and 0.98 (0.05) in men). Average step counts were in the low active range (based on 43 who provided records), average blood pressure values were close to recommended levels, and average HbA1c was above recommended targets, with one quarter on insulin. Fewer than half were ‘competent’ cooks and WEL scores indicated intermediate level ability to regulate intake in the context of food availability and negative emotions and higher ability to modulate intake in the contexts of physical discomfort, positive activities, and social pressures. The median number of classes attended was 13.

### Changes during intervention

There was a net weight reduction (mean weight change −2.2%; 95% CI −3.6 to −0.8; Table 3) overall, and this remained conclusive when the baseline values were carried forward (mean change −1.6%, 95% CI −2.6 to −0.6) as well as when the last in-session observation was carried forward (mean change −1.9%, 95% CI −3.1 to −0.7). A net weight loss of at least 5% was achieved by nearly one fifth (18.9%, 95% CI: 9.4 to 32.0) of those who completed study procedures (Table 3). Close to one third (28.6%, 95% CI 15 to 46) of those who attended at least 75% of the scheduled sessions achieved a 5% or greater net weight reduction. There were no conclusive changes in waist/hip or waist circumference.

**Table 3:** Changes from baseline to the final assessment (n=53)

		**95% CI [**^**i**^**]**
**Changes in Clinical Measures**		
≥5% weight loss, % (No.)	18.9 (10)	9.4, 32
Percent weight change, Mean (SD)	−2.2 (5.1)	−3.6, -0.8
BMI, kg/m^2b^, Mean (SD)	−0.9 (1.8)	−1.3, -0.4
Systolic blood pressure, mm Hg, Mean (SD)	−3.5 (15.8)	−7.8, 0.9
Diastolic blood pressure, mm Hg, Mean (SD)	−0.1 (10.4)	−3.0, 2.8
HbA1c, %, Mean (SD) [^j^]	−0.3 (1.0)	−0.6, -0.1
**Changes in Dietary Intakes**		
Total energy, kcal/day, Mean (SD)	−239 (612)	−408, -70
Protein, % of total energy intake, [^k^] Mean (SD)	0.8 (3.7)	−0.2, 1.8
Carbohydrate, % of total energy intake^c^, Mean (SD)	−2.1 (7.4)	−4.2, -0.1
Fat, % of total energy intake^c^, Mean (SD)	1.2 (7.2)	−0.8, 3.2
Saturated fat, % of total energy intake^3^, Mean (SD)	0.5 (2.6)	−0.2, 1.3
Fibre, g/day, Mean (SD)	−1.8 (7.3)	−3.8, 0.2
Sodium, mg/day, Mean (SD)	−384 (951)	−646, -122
**Changes in Step Counts**		
Step Counts, steps/day, Mean (SD)^a^	869 (2180)	198, 1540
**Changes in Psychobehavioural Characteristics**		
*Weight Efficacy Life-Style Scores, [*^*l*^*] Mean (SD)*		
Availability Subscale	2.3 (6.9)	0.4, 4.2
Negative Emotions Subscale	2.7 (6.6)	0.9, 4.5
Physical Discomfort Subscale	2.1 (5.4)	0.6, 3.6
Positive Activities Subscale	2.3 (5.2)	0.8, 3.7
Social Pressure Subscale	1.9 (6.0)	0.3, 3.6
Total WEL Score	11.2 (23.7)	4.7, 17.8
*Hours/day spent cooking and washing up, Mean (SD)*		
Weekdays	0.5 (1.7)	0.03, 1.0
Weekend days	0.04 (1.3)	−0.3, 0.4
Frequency of eating out (days/week), Mean (SD)^1^	0.1 (1.0)	−0.3, 0.4

^i^ 95% confidence intervals.

^j^. Change in HbA1c was based on a sample size of 51; Change in step counts was based on a sample size of 43 and represented the difference between the average steps per day taken during weeks 13 to 15 and the average steps per day taken during weeks 1 to 3; Change in frequency of eating out was based on a sample size of 33 and represented the difference between the average percentage of days eating out in a week during weeks 13 to 15 and the average percentage of days eating out in a week during weeks 1 to 3.

^k^. Assuming that 1 gram of protein equals 4 kcal, 1 gram of carbohydrates equals 4 kcal, 1 gram of total fat equals 9 kcal and 1 gram of saturated fat equals 9 kcal.

^l^ A higher score is indicative of a greater ability to resist eating under the given condition. WEL subscale scores range from 0 to 36. The Total WEL Score ranges from 0 to 180.

There were reductions in total energy (−239 kcal/day, 95% CI −408 to −70) and salt intake (mean change −384 mg/day, 95% CI −646 to −122). There was a 30-minute increase in time dedicated to meal preparation on weekdays. Eating-out frequency did not change, remaining at an average of twice per week. There was an important increase in both overall (mean change 11.2, 95% CI 4.7 to 17.8) and subscale WEL scores, indicating better ability to control eating behaviours.

A total of 43 individuals provided the records needed to compute step count changes. In these individuals, daily step counts increased by 869 (95% CI 198 to 1,540), with greater increases in those who started the intervention in the fall/winter and ended in the spring/summer (1,366 steps/day increase) compared to those who did the reverse (436 steps/day increase).

HbA1c values decreased overall, with an average absolute change of −0.3% (95% CI −0.6 to −0.1). There was a signal for a SBP reduction (−3.5 mm Hg, 95% CI −7.8 to 0.9), but there did not appear to be an important change in DBP (−0.1 mm Hg, 95% CI −3.0 to 2.8). Mean changes in HbA1c, SBP and DBP were more apparent in those who entered the study in fall/winter (−0.5% and −8.7/-1.9 mm Hg, respectively) than in those who entered in the spring/summer (−0.2% and 1.2/1.5 mm Hg, respectively).

### Associations of changes in weight and step counts with Hba1c and blood pressure

Changes in HbA1c were linked to changes in weight (Table 4) but not to changes in step count (Table 5). Across the models examined, a 2.5% weight reduction, close to the average weight change in this study, was associated with a 0.3% absolute reduction in HbA1c (Table 4; e.g., HbA1c change −0.3%, 95% CI −0.4% to −0.2% in model adjusted for age, sex,ethnicity, insulin use, and season). This was similar to the actual reductions observed (Table 3). These values were not appreciably altered by adjustment for change in step counts. An identical estimate was derived when analyses were restricted to those without any changes in antihyperglycemic medications (Table 4).

**Table 4:** Associations of weight change during intervention with changes in blood pressure and HbA1c

	**Weight Loss Increment**		
	**n**	**−2.5%**	**95% CI [**^**m**^**]**
**All with final assessment**			
*Change in systolic blood pressure, mm Hg*			
Weight change	53	−3.4	−5.4, -1.5
Weight change + age + sex + ethnicity + insulin use + season	52	−3.5	−5.5, -1.4
Weight change + step change + age + sex + ethnicity + season	41	−4.1	−6.1, -2.2
*Change in diastolic blood pressure, mm Hg*			
Weight change	53	−2.5	−3.7, -1.2
Weight change + age + sex + ethnicity + insulin use + season	52	−2.3	−3.6, -0.9
Weight change + step change + age + sex + ethnicity + season	41	−2.5	−3.9, -1.0
*Change in HbA1c, %*			
Weight change	51	−0.3	−0.4, -0.2
Weight change + age + sex + ethnicity + insulin use + season	50	−0.3	−0.4, -0.2
Weight change + step change + age + sex + ethnicity + season	39	−0.3	−0.4, -0.2
**Without antihypertensive medication changes**			
*Change in systolic blood pressure, mm Hg*			
Weight change	42	−3.2	−5.5, -1.0
Weight change + age + sex + ethnicity + insulin use + season	42	−3.5	−5.9, -1.1
Weight change + step change + age + sex + ethnicity + season	31	−4.2	−6.5, -2.0
*Change in diastolic blood pressure, mm Hg*			
Weight change	42	−2.4	−3.8, -1.0
Weight change + age + sex + ethnicity + insulin use + season	42	−2.2	−3.8, -0.6
Weight change + step change + age + sex + ethnicity + season	31	−2.4	−4.1, -0.7
**Without antihyperglycemic medication changes**			
Change in HbA1c, %			
Weight change	32	−0.3	−0.4, -0.2
Weight change + age + sex + ethnicity + insulin use + season	32	−0.3	−0.4, -0.2
Weight change + step change + age + sex + ethnicity + season	25	−0.3	−0.4, -0.2

^m^ 95% confidence intervals.

**Table 5:** **Associations of step count change during intervention with changes in blood pressure and HbA1c [**^**n**^**]**

	**Step Count Increment**		
	**n**	**1,000**	95% CI^*^
**All with final assessment**			
*Change in systolic blood pressure, mm Hg*			
Step count change	41	−2.9	−4.9, -0.8
Step count change + age + sex + ethnicity + insulin use + season	40	−2.3	−4.5, -0.1
Step count change + weight change + age + sex + ethnicity + season	41	−1.4	−3.2, 0.4
*Change in diastolic blood pressure, mm Hg*			
Step count change	41	−1.8	−3.2, -0.4
Step count change + age + sex + ethnicity + insulin use + season	40	−1.6	−3.1, -0.2
Step count change + weight change + age + sex + ethnicity + season	41	−1.2	−2.6, 0.2
*Change in HbA1c,*^%^			
Step count change	39	−0.1	−0.2, 0.1
Step count change + age + sex + ethnicity + insulin use + season	38	−0.1	−0.2, 0.1
Step count change + weight change + age + sex + ethnicity + season	39	0.01	−0.1, 0.1
**Without antihypertensive medication changes**			
*Change in systolic blood pressure, mm Hg*			
Step count change	31	−2.5	−4.7, -0.3
Step count change + age + sex + ethnicity + insulin use + season	31	−2.2	−4.6, 0.2
Step count change + weight change + age + sex + ethnicity + season	31	−1.4	−3.3, 0.6
*Change in diastolic blood pressure, mm Hg*			
Step count change	31	−1.5	−0.3, 0.03
Step count change + age + sex + ethnicity + insulin use + season	31	−1.5	−3.1, 0.2
Step count change + weight change + age + sex + ethnicity + season	31	−1.0	−2.5, 0.5
**Without antihyperglycemic medication changes**			
*Change in HbA1c,%*			
Step count change	25	−0.03	−0.1, 0.1
Step count change + age + sex + ethnicity + insulin use + season	25	−0.01	−0.2, 0.1
Step count change + weight change + age + sex + ethnicity + season	25	−0.001	−0.1, 0.1

^n^ Change in step counts represented the difference between the average steps per day taken during weeks 13 to 15 and the average steps per day taken during weeks 1 to 3. Although 43 individuals provided step count records required, 2 among these experienced technical difficulties during blood pressure assessment.

Changes in both SBP and DBP were related to changes in weight (Table 4). After adjustment for age, sex, ethnicity, insulin use, and season, a 2.5% weight reduction was associated with a −3.5/-2.3 mm Hg mean change in SBP/DBP (95% CI −5.5 to −1.4/-3.6 to −0.9). These values were not appreciably altered by adjustment for change in step counts. In contrast, although changes in both SBP and DBP also appeared to be related to changes in step count (Table 5), the magnitude of these associations declined with adjustment for weight change and an absence of association could not be excluded. A 1,000 step per day increment was associated with a −2.3/-1.6 mm Hg mean change in SBP/DBP in models adjusted for age, sex, ethnicity, insulin use, and season (95% CI-4.5 to −0.1/-3.1 to −0.2) but with adjustment for weight change, these values were −1.4/-1.2 (95% CI −3.2 to 0.4/-2.6 to 0.2) Changes in salt intake were not linked to changes in blood pressure levels. When analyses were restricted to individuals without changes in antihypertensive medications (Tables 4 and 5), the point estimates remained similar.

## Discussion

Among adults with DM2, we conducted a phase 2 trial to assess the effects of a 24- week 15- core session nutritional education program that focused on meal preparation training and integrated pedometer-based self-monitoring. In the 74% of participants who completed assessments, our findings demonstrate small but conclusive reductions in weight (mean weight change −2.2%; 95% CI −3.6 to −0.8) with a nearly 1- point decrease in BMI (mean change −0.9, 95% CI −1.3 to −0.4) and a net increase in step counts (mean change 869 steps/day, 95% CI, 198 to 1,540) in those who provided step count records. Although eating- out frequency did not decline, there were reductions in reported energy intake and salt consumption, as well as improvements across a range of behaviours related to the ability to control eating, and increases in time dedicated to meal preparation on weekdays. Most importantly, there was a definitive improvement in glycemic control (mean HbA1c change - 0.3%, 95% CI −0.6 to −0.1) that was not attributable to medication changes, confirming the physiological impact of the intervention. Further, there was a strong suggestion of systolic blood pressure improvement (mean change −3.5 mm Hg, 95% CI −7.8 to 0.9). We believe that these findings indicate that the intervention holds promise and merits further development and testing.

We acknowledge that the weight loss effects that we observed are less than reported in some other intervention studies. Intervention studies that have focused primarily on nondiabetic individuals have achieved a net reduction of 5% or greater in a larger proportion of participants [6,31-33] compared to the one fifth of our study participants. This is likely partly attributable to our focus on individuals with DM2, perhaps underscoring the particular challenge that weight loss poses to DM2 patients [14].

Impressively, and specifically in adults with DM2, the Look AHEAD trial achieved a 5% or greater weight loss in roughly 70% of participants at one year [34]. The most notable difference between our strategy and that employed in Look AHEAD is the use of meal replacements in Look AHEAD [35]. During the first 20 weeks of the Look AHEAD intervention, participants are prescribed two meal replacements per day, one portion- controlled snack, and one self- selected meal. After 20 weeks, they are prescribed one meal replacement and two meals of self-selected foods, and use of weight- lowering medication is considered if weight loss is deemed insufficient. The use of meal replacements has been demonstrated to be an important predictor of weight loss in the Look AHEAD cohort [15]. However, it does potentially limit variety and flexibility in dietary intake and may therefore not be uniformly accepted, hence the need for the development of alternative strategies. Additionally, the long term feasibility of sustained used of meal replacements has not been reported; there is a very real possibility that while meal replacements may lead to greater initial weight loss, once these are discontinued, this benefit may not persist. This underscores the need for studies such as ours that endeavour to develop and test alternatives to meal replacement-based strategies.

It should also be noted that in Look AHEAD failure to complete the run- in for dietary intake and exercise was an exclusion criterion; [35] we did not use a run-in period. This may also have contributed to the lesser degree of weight loss observed in our study.

In our strategy, by integrating step count self- monitoring, study participants who provided step count records achieved an 869 average increase in daily step counts, roughly one third that achieved in interventions that focus primarily on increasing walking and other step- related activity [22]. Given that the main focus of the intervention was on nutritional issues, the increase in step counts was notable. The step count increase was greater in those who started the intervention in fall/winter and ended in spring/summer compared to those who did the opposite (1,366 vs.436 steps/day). This is consistent with a previous observational study wherein we demonstrated that during fall/winter, step counts decline by 758 steps/day [21]. Thus, in the present study, those who started the intervention during the spring/summer were not only able to offset the usual fall/winter decline, they were also able to achieve a net increase. Arguably, a mean increase of at least1,000 steps/day may be needed to demonstrate definite health effects, as supported by our own observational studies [7,8]. Nonetheless, the 869 steps/day increase is promising, and could potentially rise to 1,000 steps/day or more with greater focus on this aspect of behavioural change.

A key finding was the demonstration of a definitive 0.3% reduction in HbA1c that was closely associated with the weight changes observed (Table 4). This reduction was determined to be unrelated to changes in antihyperglycemic agents. In terms of magnitude, it is roughly one third of the HbA1c reduction in the intensive glycemic control with metformin arm of the United Kingdom Prospective Diabetes Study (UKPDS), an arm with a 42% relative reduction in diabetes- related deaths. [36] Based on UKPDS analyses, Tricco and colleagues [37] recently estimated that a 0.33% HbA1c reduction may be associated with 21% fewer deaths, 14% fewer myocardial infarctions, and 37% fewer microvascular complications (retinopathy, nephropathy, neuropathy) at the population level. This estimate supports the clinical importance of the 0.3% HbA1c reduction that we observed.

Thus, even with a mean weight reduction of less than 5%, our intervention demonstrated clinically- important improvements in glycemic control. Such physiological effects of small weight changes have been demonstrated in other DM2 intervention trials, including the group that had a net weight change of 2 to 5% over a one-year period in the Look AHEAD trial [2].

Another important signal of potential for physiological impact was the SBP reduction, although this was not definitive (95% mean change – 3.5 mm Hg, 95% CI −7.8 to 0.9), likely because of insufficient sample size. In regression analyses, however, definitive association with weight change were demonstrated: a −2.5% weight change was associated with a 3.5 mm Hg SBP reduction (95% CI −5.5 to −1.4). There was also a signal that in some individuals the increase in step counts may have contributed to blood pressure reductions; however, this was not clearly independent of weight change, and was not the primary focus of the strategy. Greater emphasis on methods to increase step counts may enhance the impact of this aspect of the intervention. For example, a recent trial suggests that encouraging stepping during television commercial breaks may importantly increase daily steps [38].

Both participants who began the 24-week intervention in the spring/summer and those who started in the fall/winter experienced HbA1c reductions (−0.2% and −0.5%, respectively). Blood pressure– lowering was apparent for those who started in the fall/winter (SBP/DBP −8.7/-1.9 mm Hg) but those who started in the spring/summer experienced a small increase (1.2/1.5 mmHg): this must be interpreted, however, in the context of a usual fall/winter 4 mmHg SBP increase, as we have previously documented in this patient population [21]. Thus the intervention appears to have attenuated the usual fall/winter increase.

The main limitations of our study are related to sample size and use of a single-arm intervention rather than a randomized controlled design. We would emphasize, however, that a single- arm trial- i.e., a Phase2 trial- was deliberately pursued in order to assess for feasibility and evidence of some impact. A single-arm design permitted each participant to act as his/her own control, reducing confounding and increasing the precision of estimates. In contrast, small trials risk unbalanced treatment arms and limited scope for statistical adjustments. Another potential limitation relates to use of participant reports to estimate dietary intake and step counts. While we did detect reductions in energy intake and salt intake as well as increases in step counts (Table 3), we acknowledge that self-report of dietary intake may be subject to either over or under reporting as it relies on an individual’s memory and ability to estimate portion sizes. [39] Similarly, use of step count records to monitor physical activity requires participants to reliably wear a pedometer and accurately record values. Indeed, not all of those who completed final assessments reliably provided step count records. Thus, both the estimates of dietary intake and step counts are subject to some degree of measurement error. We would emphasize, however, that the main parameters of interest in this study- changes in weight, blood pressure, and HbA1c- were all objectively-assessed.

Despite some potential limitations, the findings provide good justification for further development and ultimately testing of this innovative strategy through a large randomized controlled trial. Importantly, such a trial should be of sufficient duration to allow examination of longer term effects, adherence, and sustainability (i.e., one year and beyond). Indeed we are presently attempting to tailor a similar strategy to women with a history of gestational diabetes mellitus (Canadian Institutes of Health Research, Principal Investigator K. Dasgupta, CAI 117789); this younger patient population at risk for DM2 may be able to achieve greater changes in weight and step counts, potentially translating into even greater vascular health improvements. Further, contingent on focus group discussions, we are considering the implementation of a tapering course of meal replacements *concurrent* with the acquisition of meal preparation skills. This could diminish long term reliance on meal replacement products, while allowing initial benefit from their use.

In this study, the average cost per participant per session (and covered through the study) was roughly $50 CDN (room rental, chef, groceries, dietitian), with a 15- session program costing approximately $750.00 CDN per participant. In an actual program, such a fee could be prohibitively high for some persons with DM2, given the higher prevalence of DM2 in more disadvantaged populations [40]. Translating such a strategy into a sustainable program will thus require important cooperation/contributions from key stakeholders (diabetes associations, local businesses, government agencies). While sessions were held at kitchen workshops of a grocery chain, other options would include kitchens at schools and community centres.

The findings reported in this study provide evidence to support the merits of a group-based dietary education strategy that is centered on preparing a meal, eating it together, and discussing both optimal eating habits and the challenges of adopting these. In individuals followed for DM2, the strategy tested demonstrated reductions in weight as well as effects on glycemic and blood pressure control- even in the context of medication therapy. There is a need to further develop and target the intervention to achieve a greater magnitude of effect. Nonetheless, our findings support the merit of this line of enquiry. The prevention of overweight- related complications requires a range of creative management options, allowing a ‘fit’ between patient and approach. Our strategy may emerge as one such option, empowering overweight persons with or at- risk for DM2 with the tools to develop a healthy relationship with food and better physical activity habits in the modern obesogenic environment.

## Abbreviations

DM2: Type 2 diabetes mellitus; HbA1c: Glycosylated hemoglobin; WEL: Weight efficacy lifestyle (Questionnaire); MI: Myocardial infarction; BMI: Body mass index; CDA: Canadian diabetes association; SBP: Systemic blood pressure; DBP: Diastolic blood pressure; SD: Standard deviation; CI: Confidence Interval.

## Competing interests

The author(s) declare that they have no competing interests.

## Authors’ contributions

KD, RG, LJ, and DD designed research; KD and RG conducted research; SH and LJ analyzed data; KD wrote the paper; SC and RG interpreted and edited the paper; KD had primary responsibility for final content. All authors read and approved the final manuscript.

## Authors’ information

Kaberi Dasgupta is a Physician Scientist and Associate Professor of Medicine at McGill University. Samantha Hajna is a doctoral student in Epidemiology at McGill University. Lawrence Joseph is a Professor of Medicine at McGill University and a senior biostatistician. Deborah Da Costa is Associate Professor of Medicine at McGill University and a clinical psychologist and researcher. Stavroula Christopoulos is an endocrinologist at the Sir Mortimer Davis Jewish General Hospital in Montreal. Rejeanne Gougeon is Associate Professor of Medicine at McGill University and an expert in Human Nutrition. She has led the development of the nutrition guidelines of the Canadian Diabetes Association.
